# Lipid-Enriched Parenteral Nutrition and Bloodstream Infections in Hospitalized Patients: Is It a Real Concern?

**DOI:** 10.3390/medicina58070885

**Published:** 2022-07-01

**Authors:** Antonio Tota, Amato Serra, Pauline Raoul, Antonio Gasbarrini, Emanuele Rinninella, Maria Cristina Mele

**Affiliations:** 1 Scuola di Specializzazione in Medicina Interna, Università Cattolica del Sacro Cuore, Sede di Roma, Largo F. Vito, 00168 Rome, Italy; antonio.tota1991@gmail.com (A.T.); amatoserra@gmail.com (A.S.); 2 UOC di Nutrizione Clinica, Dipartimento di Scienze Mediche e Chirurgiche, Fondazione Policlinico Universitario A. Gemelli IRCCS, Largo A. Gemelli 8, 00168 Rome, Italy; paulineceline.raoul@policlinicogemelli.it (P.R.); mariacristina.mele@unicatt.it (M.C.M.); 3 UOC di Medicina Interna e Gastroenterologia, Dipartimento di Scienze Mediche e Chirurgiche, Fondazione Policlinico Universitario A. Gemelli IRCCS, Largo A. Gemelli 8, 00168 Rome, Italy; antonio.gasbarrini@unicatt.it; 4 Dipartimento di Medicina e Chirurgia Traslazionale, Università Cattolica del Sacro Cuore, Sede di Roma, Largo F. Vito 1, 00168 Rome, Italy

**Keywords:** clinical practice, fatty acids, infectious complications, lipids, nutritional support, omega-3 fatty acids, parenteral nutrition, sepsis

## Abstract

Today, few clinicians are still convinced that lipids are sepsis risk factors in patients receiving parenteral nutrition. This dogma is principally based on old literature. This review deals with the most recent literature search that provided up-to-date data over the past ten years. Systematic research was performed on Pubmed, MEDLINE, and Web of Science. The recent evidence does not justify the exclusion of lipid emulsions in patients receiving parenteral nutrition for fear of bloodstream infection risk. Moreover, lipids represent a substantial proportion of the energy source providing essential fatty acids, potentially improving clinical outcomes in patients often malnourished. Understanding the actual risk factors of sepsis during parenteral nutrition is necessary to optimize patient nutritional status and care and avoid essential fatty acid deficiency. There is an urgent need to make updated nutrition training available at all levels of medical education.

## 1. Introduction

Parenteral nutrition (PN) is the means by which nutrients are provided intravenously. It can be administered via a catheter with the tip located in a central vein—central PN—or via a cannula inserted into a peripheral vein—peripheral PN—depending on the osmolarity of the mixture. For patients for whom central catheterization is impossible, PN can also be administered via an arteriovenous shunt.

PN is recommended in malnourished patients or those at risk of malnutrition in some conditions—such as gut functional unavailability or gut physical inaccessibility [1]. Central PN may be associated with catheter-related bloodstream infections (CRBSIs). Indeed, an intravenous line can be colonized by bacteria or fungi, potentially seeding into the bloodstream. CRBSIs are associated with high morbidity and mortality [2]. Multiple mechanisms are known to increase the risk of infectious complications in patients receiving central or peripheral PN: poor central venous catheter care, contamination of the cannula and the cannula wound, infusate contamination, or colonization of other parts of the PN system by some varieties of microorganisms (bacteria and fungi) such as *Candida albicans* (*C. Albicans*). Enteral starvation may concur to increase this risk due to an impairment of the gut barrier and bacterial translocation [3]. Among many clinicians, there is a concern that adding lipid emulsions to PN could cause fungal infections [4]. This concern has spread across the decades among clinicians as a dogma. Hence, some of them still now exclude lipids emulsions (LEs) regardless of the nutritional status of patients. This review aims to gather the latest data about the sepsis risk of LEs in PN and demonstrate to clinicians the safety of LE addition and their importance as a primary source of essential fatty acids in PN in hospitalized patients.

## 2. Materials and Methods

An evidence-based approach from the scientific literature through systematic research was used on Pubmed, MEDLINE, and Web of Science. MeSH terms ((intravenous lipid emulsions [MeSH Terms]) AND ((sepsis [MeSH Terms]) OR (catheter-related infection [MeSH Terms])) were used to find related articles from inception to date. All abstracts were read, and irrelevant articles were excluded. Additional articles were retrieved from references’ list of relevant papers.

## 3. Parenteral Nutrition and Sepsis

### 3.1. Generalities on Sepsis

Sepsis is a potentially life-threatening condition characterized by organ dysfunction caused by a dysregulated host response to infection. Septic shock is a complication of sepsis with circulatory and cellular/metabolic dysfunction, and thus is associated with higher mortality risk. Sepsis is associated with increased morbidity and mortality [2] and is considered a significant healthcare problem, affecting millions of people worldwide each year. This condition has a mortality risk ranging from moderate (e.g., 10%) to substantial (e.g., >40%) depending on the etiologic agent, biological characteristics of the host, the timeliness of recognition, and provision of adequate therapy [5]. Concerning its pathophysiology, sepsis occurs when the response to an infection causes the release of proinflammatory molecules, the so-called cytokine storm, that exceeds the boundaries of the local environment, leading to a more systemic reaction [6]. Most cases of sepsis are triggered by hospital-acquired Gram-negative bacilli or Gram-positive cocci and often occur in immunocompromised patients or patients affected by chronic diseases. Infrequently, it is caused by *Candida* or other fungi. Current therapies consist of adequate antibiotics/antifungal agents targeting the underlying infection, fluid repletion to improve stroke volume, vasopressors if needed in refractory hypotension, and high-quality supportive care [7]. 

Sepsis can be a complication of parenteral nutrition, as a consequence of phlebitis, the most common complication of peripheral parenteral nutrition, or as a complication of central venous catheters (CVCs) used for central parenteral nutrition. Usually, we refer to the septic complications of intravascular catheters with the term CRBSI. Bacteria or fungi can colonize a catheter, and when they grow significantly, they seed the systemic circulation causing clinical signs of infection to become apparent. These signs cover a broad spectrum from subfebrile status up to symptoms of MOF and septic shock. It has also been hypothesized that TPN, by-passing the intestinal tract, causes mucosal gut atrophy and dysbiosis, and increases bacterial translocation thus promoting sepsis [8].

### 3.2. Parenteral Nutrition and Infectious Complications: A « Liaison Dangereuse »

The concept of peripheral parenteral nutrition (PPN) was first depicted by Brunschwig, in 1945. Together with his colleagues, he fed a patient suffering from multiple enteral fistulas, for eight weeks, using a protein hydrolysate and a 10% dextrose solution. Later, in the 1950s and 1960s, an amino acid solution and lipid emulsion were first introduced, respectively [9,10].

In the early days of PN in the United States, glucose and amino acids solutions with all other nutrient components were mixed, initially by the physicians and/or nurses, in 1-L glass bottles; then, a short time later, by pharmacists in more extensive (1–2 l) glass bottles and eventually in PVC bags. Fat emulsions, when given, were infused separately through Y-connectors, and this method was later called “2 + 1” [11].

On the other hand, in Europe, a multiple (double or triple) bottle (MB) system was used, in which amino acid, glucose, and lipids were administered in parallel or sequence using multiple different bottles. It was common at that time to change the bottle with the preparation for PN up to 6–8 times a day, to make many other additions, and numerous peripheral vein punctures, thus increasing the risk of solution administration errors, hyperglycemia, electrolyte disorders, and infectious complications.

Nowadays, the All-in-One (AIO, 3-in-1) system incorporates all the elements of PN mixed in one container [12]. This system has many advantages, among which is a reduced risk of contamination of the infusion system by multiple manipulations and interventions. Furthermore, due to fewer connections and bottle changes, the use of a closed system, the rate of sepsis is decreased [13].

Since its birth, PN has been related to the possibility of metabolic, mechanical, and infectious complications. PN is often considered an ideal microbial growth medium, since its slow administration at room temperature permits microbes to proliferate and cause adverse effects [14].

The risk of infectious complications in patients receiving PN is related to multiple mechanisms: central venous catheter care, contamination at different levels (cannula, infusate, other parts of the PN system), and cannula wound infection. This results in various microorganisms (bacteria and fungi) potentially associated with infectious complications

Multiple approaches prevent PN’s inadvertent microbial contamination and limit microbial growth, including PN preparation with an aseptic technique and reducing the times of bag changes [15].

In addition, the Healthcare Infection Control Practices Advisory Committee (HICPAC) hypothesized that lipid addition in PN constitutes a specific risk for microbial growth [16], recommending that administration sets linked to lipid-containing PN (or lipid emulsion) should be changed within 24 h after infusion beginning or up to 48 h for lipid-free PN. This recommendation was supported by the Cochrane Collaboration and the UK Epic Project after finding no evidence to challenge them [17].

The European Society for Clinical Nutrition and Metabolism (ESPEN) also considers lipid PN as an infection risk factor if administration sets are used beyond 24 h [18].

These recommendations have been applied in clinical practice by restricting the administration of lipid PN from a single bag to no more than 24 h, even in favorable conditions, such as when starting PN or weaning from PN, when it might have been infused over more prolonged periods [1].

Recommendations reported above are supported by evidence derived from a few studies, of variable quality, which sometimes did not compare lipid and lipid-free PN, sometimes reporting contradictory results and giving little or no regard to confounding variables or methodologies used. Furthermore, other recommendations are based on expert opinion alone.

Major clinical nutrition societies never stated that administering PN without the lipidic component could reduce the rate of infectious complications related to PN [19].

Contrariwise, in the standard clinical practice, many clinicians prefer administering the PN solution without the lipidic component, especially in septic patients, those at risk of infectious complications, or even those without particular risk factors. This practice is supported by the belief, not validated by any scientific evidence, that the lipid component of PN increases the rate of infectious complications and sepsis above all [14].

## 4. Lipids in Parenteral Nutrition: Biochemistry and Properties

Lipids are essential energy substrates and are the primary energy stored in our body. Moreover, phospholipids are crucial structural components of cell membranes, including the plasma membrane, vacuoles, and other organelles; fatty acids (FA) may affect cell functions and are precursors of eicosanoid synthesis, while cholesterol is a precursor for steroid hormones’ synthesis. In addition, lipids alter transcription factor activity, eicosanoids metabolism, cytokines production, and gene expression [20].

LEs provide high amounts of fuel calories and essential fatty acids, which are key components of PN regimens. Lipid intake should cover 20 to 40% of energy needs, depending on individual tolerance and clinical situation. In some patients, especially those with acute respiratory failure, in the presence of fat emulsion tolerance, up to 50% of total energy can be administered as a lipid emulsion to help maintain a usual respiratory quotient.

Intravenous lipid emulsions (IVLE) have been developed on the model of the intestinal chylomicron, with triglycerides and lipid-soluble vitamins in the core of the molecule and phospholipids, free cholesterol, and other lipid-soluble vitamins on the surface [21]. However, exogenous emulsion particles essentially differ from endogenous lipoproteins. For example, no apoproteins (apo B-48 and apo A-1) nor esterified cholesterol are contained in exogenous emulsions. The composition of their component, such as the FA pattern, differs from that of endogenous counterparts. Nonetheless, emulsion particles rapidly acquire exchangeable apoproteins (C-I, C-II, C-III, E, A-IV). At the same time, they are delivered to the bloodstream, following the same intravascular metabolic pathways of chylomicrons.

Significant differences exist between Europe and the USA regarding the use of IVLE. In the USA, the first lipid emulsions, mainly derived from cottonseed oils, were associated with side effects, so their use was limited until 1977, also explaining the low lipid content in their preparation for enteral nutrition.

Conversely, in Europe, Wretlind et al. developed a lipid emulsion based on soybean oil emulsified with egg phosphatides that was clinically well tolerated and has been widely used, not only to prevent a deficiency of essential FAs but also to supply significant energy intake (from 30 to 40% of infused calories) even in stressed patients with insulin resistance.

These “first generation” LEs contain a much higher proportion of (mainly ω-6) polyunsaturated FAs, such as linoleic acid and alfa-linolenic acid, but a relatively low amount of antioxidants such as alfa-tocopherol. Their disadvantage is that they can enhance the production of pro-inflammatory prostaglandins, leukotrienes, and thromboxanes and stimulate proinflammatory cytokine release, thus leading to inflammation [22].

In the last two decades, these unfavorable effects led to the development of the so-called “second generation” LEs. They contain a mixture of medium and long-chain triglycerides (MCT/LCT) instead of only LCT containing LEs. Medium-chain triglycerides, derived from coconut or palm kernel oil, have better solubility and are more readily hydrolyzed by lipases and more quickly taken up by peripheral tissues than LCT. Other “second generation” lipid emulsions are made of so-called “structured triglycerides,” phased out in most countries, or olive oil/soybean oil, composed of 80% olive oil and 20% soybean oil. Olive oil contains fewer ω-6 FAs and a good amount of oleic acid (mono-unsaturated ω-9 FA), which is not a precursor for pro-inflammatory prostaglandin synthesis [23]. Compared to MCT/LCT IVLEs, an international, multicenter, prospective, randomized, open-label trial by Pontes-Arruda et al. found no difference in catheter-related bloodstream infections (CRBSI) rates between patients receiving an olive oil-based IVLE versus those receiving an MCT/LCT based IVLE (25 vs. 21, respectively; *p* = 0.62) [24].

In conclusion, these “second generation” kinds of lipid emulsions appeared to be of benefit to patients with SIRS or sepsis because—containing only half the amount of LCT—they supply a significantly smaller quantity of ω-6 FAs, reducing the amount of the precursors of potentially immunosuppressive prostaglandins [25].

The newest generation of IVLEs—the “third-generation” one—includes fish oil combined with two or more oils used in the former generations of IVLEs. It is well known that ω-3 polyunsaturated FAs (eicosapentaenoic-EPA and docosahexaenoic-DCA) decrease inflammatory activity and diminish infectious complications. Fish oil contains ω-3 FAs from which less inflammatory eicosanoids are derived, compared to those originating from ω-6 FAs in soy oil [26]; moreover, they constitute a substrate for the production of Specialized Proresolving Molecules (SPMs), which play a role in the resolution of inflammatory processes [27], such as sepsis, SIRS, trauma, Intestinal Failure Associated Lived Disease (IFALD), and burns [28]. These preparations aim to provide an efficient source of energy but also at helping modulate important metabolic and immune reactions due to the rapid delivery of ω-3 FAs from lipid emulsion to tissue cell membranes [29].

An example of “third-generation” LE is a 4-oil mixed IVLEs (20%; Fresenius Kabi, Smofkabiven) containing soy oil, MCTs, olive oil, and fish oil. Its use in critically ill patients may provide clinical benefits, a concentrated energy source, and adequate essential FAs without propagating inflammation in an already inflamed population [30]. Some recent meta-analyses, including three studies and 110 patients receiving a 4-oil mixed IVFE, reported better antioxidant profiles, absence of essential FA deficiency, improved ω-6/ω-3 ratio, and better liver function tests. No substantial changes in inflammatory markers were observed [31]. In conclusion, there is growing evidence that 4-oil mixed IVFE formulations containing soy oil, fish oil, MCTs, and olive oil should be preferred over conventional soy oil-based IVFE in critically ill patients.

Other microcomponents in LEs that play some role and modify their functional characteristics are: phytosterols, α-tocopherol, emulsifiers, and vitamin K [32]. Phytosterols, structural components of biological membranes of plants, include sitosterol, campesterol, and stigmasterol; they share a similar structure with cholesterol and may be involved in the cholestatic liver disease associated with TPN. α-tocopherol, a form of vitamin E, is added to LEs to prevent the peroxidation of PUFA-rich lipids because of unsaturated double-bonds [33]. Since LEs consist of oil in water, emulsifiers such as phospholipids allow fat droplets to be dispersed in the aqueous phase. Given its importance in blood clotting, a small amount of vitamin K is added to LEs, which vary in different formulations [34].

## 5. Parenteral Nutrition in Septic Patients

During the complex metabolic response that the body processes under a critical illness such as sepsis, the increased energy requirements and the need of the immune system for the efficacy of the body’s defense are fulfilled by the diversion of energy and specific substrates, thus resulting in metabolic modifications such as increasing in gluconeogenesis and insulin resistance, among others. These life-giving responses occur at the cost of body protein loss (especially muscle, skin, gastrointestinal tissues, and even bones), and nutritional support can play a role in offsetting the negative energy and protein balance. In critically ill patients, the administration of lipids is a fundamental part of nutritional support: they are primary energy substrates for hepatocytes, myocardium, and skeletal muscle, considering that the FAs turnover is augmented in critical conditions [35].

As well known, enteral nutrition shows beneficial effects on gastrointestinal and immune systems. For these reasons, a portion of nutritional intake should preferably be administered enterally, while supplemental parenteral nutrition should be used in the presence of protracted gastrointestinal dysfunction in the recovery phase.

In a prospective, single-center, observational cohort study, Ulusoy et al. enrolled septic patients that needed parenteral nutrition, which was administrated with soy-bean oil-based or olive oil-based parenteral lipid emulsions for ten days or more, and they determined adipokine and cytokine concentrations at enrollment and ten days after. A significant decrease in serum leptin, resistin, and cytokines (IL-6, IL-10, IL-1β, and TNF-α) levels was found in the whole study population over ten days following sepsis (*p* < 0.05). Furthermore, they demonstrated an association between percentage changes in adiponectin, resistin, and visfatin concentrations and survival (log-rank test: *p* < 0.05) [36]. So, it can be argued that adipokines may play a role as functional prognostic biomarkers in critically ill patients with sepsis.

Nevertheless, conventional soybean LEs contain excessive polyunsaturated FAs and insufficient amounts of alfa-tocopherol. Their use can be associated with increased production of peroxidative metabolites and an excess of ω-6 (especially linoleic acid). This can result in high levels of pro-inflammatory leukotrienes and prostaglandins, thus depressing immune defense and augmenting the systemic inflammatory response [22].

Similarly, “second generation” LEs, enriched with elevated levels of MCT, can lead to an increased metabolic requirement because of higher TEN (Thermic Effect of Nutrition, a phenomenon for which energy intake exceeding energy demands leads to a further elevation in energy expenditure). On the contrary, emulsions which are containing LCT cause only a small TEN (2–3%) [25].

There is growing evidence that “third-generation” LEs, the latest and most common, including 4-oil mixed IVFE, should be selected, when necessary, over other types of IVLEs in critically ill patients since they could provide benefits over inflammatory status. Two meta-analyses in 2010 showed positive effects of LEs administration on clinical outcomes of gastrointestinal surgical patients. They compared soybean oil-LE and fish oil-LE, founding that fish-oil LEs were generally well tolerated and safe with decreased infectious complications (OR = 0.56, *p* = 0.04) and shorter length of stay in hospital and ICU (weighted mean difference = −2.98, *p* < 0.001) [22]. A work corroborated this evidence by Grau-Carmona et al. demonstrated that critically ill patients, receiving ω-3 based LEs, had a lower rate of hospital-acquired infections compared with administration of fish-oil free LEs (21.0% vs. 37.2%, *p* = 0.035), thus strengthening the evidence of the anti-inflammatory effect of this kind of fatty acids [37].

## 6. Lipid Emulsions and Infectious Complications

Several theories have been proposed to explain the supposed implication of IVLEs in increasing infectious complications, including CRBSI.

IVLEs are alkaline and isotonic to the plasma, constituting an optimal growth pabulum for microbes, so it has been supposed that the lipid dose and administration rate may compromise the function of the reticuloendothelial system, leading to competition between lipid and microorganisms for clearance. This would result in a hepatic deposition of LCT that may determine a decreased ability of Kupfer cells to free hepatic toxins, including harmful microbes [38]. IVFEs have a plasma clearance that is influenced by particle size, phospholipid content, and infusion rate [39]. Faster administration rates and larger particles can determine an excessive uptake by the reticuloendothelial system, causing functional impairment in clearing microbes [40].

The first LE considered safe for PN was based on soybean oil (“Intralipid”), developed in 1961 in Sweden by A. Wretlind, as seen before, and its use allowed a drastic reduction in complications of high-dose dextrose infusions reported in America with the use of lipid-free intravenous hyperalimentation [41]. A case report by McKee et al. in 1979 described two cases of Gram-negative sepsis associated with the use of Intralipid, likelihood due to instances of extrinsic contamination of the LE. The authors concluded that not only the total parenteral nutrition (containing a mixture of amino acids, glucose, electrolytes, and vitamins), but also LEs were associated with the risk of sepsis [42].

Other works analyzed, dating from the early 1970s to late 1980s, stated that IVLEs, especially soybean and safflower oil emulsions, if contaminated, can support the growth of various Gram-positive and Gram-negative bacteria and several species of *Candida* [43].

A 1985 review by W. Williams, “Infection control during parenteral nutrition therapy”, stated that the exact mechanism that initiates infectious complications, such as cannula-related infection, is poorly understood, nonetheless, multiple factors may have a role: predisposition of the host, cannula material, its insertion technique and site of insertion, colonization of PN cannulas, use of PN systems for multiple purposes. The author concluded that many facets of PN therapy were based on data from uncontrolled clinical investigations and that controlled clinical trials would have provided more strict data that would have further minimized the risks associated with PN [44]. No data emerged regarding an increase in infectious risk, linked specifically to the lipidic component.

In the early 1990s, a prospective randomized trial by D’Angio et al., in which patients receiving PN were randomized in two different groups differing in the mode of administration of lipids—with other PN macromolecules in a TNA (Total Nutrient Admixture) or separately via intravenous piggyback (PB)—concluded that use of TNAs did not influence the rate of infections, since there was not a statistically significative difference in the incidence of infections between the two groups (12.6 and 10.3 per 1000 days of PN in the TNA and PB groups, respectively; *p* = 0.89) [45].

Two other prospective trials of the late 1990s suggested that the early discontinuation of IVLE during PN might be associated with better clinical outcomes in critically ill patients. In one of them, Battistella et al. demonstrated an association between withholding IVLE for 10 days and lower rates of infectious diseases, such as pneumonia (73% early IVLE vs. 48.1% delayed IVLE) and catheter-related bloodstream infections (43.3% vs. 18.5%) in patients hospitalized for trauma [46]. Similarly, McCowen et al. evaluated 40 medical/surgical adult patients hospitalized in intensive care units and compared standard PN with IVLE vs. hypocaloric PN without IVLE. The study resulted in fewer infections in the group who received PN without IVLE (47.6% vs. 31.6%) [47].

Gerlach et al. later disproved these findings in a retrospective study on surgical intensive care unit patients. He found that withholding IVLE therapy during the first seven to ten days of PN did not impact the risk of infectious complications (63.3% having immediate IVFE vs. 67.6% having delayed IVFE; *p* = 0.79) or the mortality rate (63.3% vs. 55.9%, respectively; *p* = 0.63) [40]. Similar findings were derived from a retrospective study by Cheng et al., which assessed the rate of infections in hospitalized adult patients who received nondaily or, alternatively, daily PN with IVLE. Though limited by the small sample size in analysis, they found that nondaily IVLE infusion did not significantly influence the risk of infection or disease development time (45.28 vs. 21.24 total cases of infections per 1000 catheter days, *p* = 0.203; 11.24 vs. 6.59 days to first infection, *p* = 0.30) compared with daily IVLE administration [25].

Another important study that showed no link between LEs and infectious com-plications rate was performed by Pontes-Arruda et al. [24]. The trial evaluated the overall bacterial and bloodstream infection rates in over 4000 patients receiving PN with or without lipid emulsion. A lower number of cases of both overall bacterial infection (43.5% vs. 53.5%) and bloodstream infection (14.5% vs. 18.9%) was found in the group with lipids-free PN. However, after correcting for baseline characteristics, the study revealed no statistically significant differences in overall risk of bacterial infections (51.4% vs. 53.5%; odds ratio [OR] = 1.11; 95% confidence interval [CI], 0.96–1.27) or bloodstream infections (19.6% vs. 19.2%; 0.97; 0.81–1.16) between the two groups. Similarly, in the evaluation of patients hospitalized in the intensive care unit for more than three days, they found fewer bacterial infectious complications (58.3% vs. 67.3%) and bloodstream infections (31.0% vs. 37.0%) in the lipids-free group. However, even in this case, after adjustment for baseline characteristics, no significant differences in risk of overall bacterial infection (OR = 0.95; 95% CI, 0.75–1.22) or bloodstream infection (OR = 0.92; 0.71–1.19) were found between the two groups. In conclusion, the authors inferred that the risk of developing infectious complications, for patients receiving PN, was not related to the infusion of lipids (soybean oil-derived, in this case) [24].

A review and meta-analyses by Austin [14] were performed to evaluate the possible role of LE in influencing microbial growth in PN. They showed large variability in microbial growth, which was influenced by microbial species. In fact, it was proved that some microbial species (for example, *C. albicans* e *S. marcescens*) had rapid growth in both lipid and lipid-free PN, while other microbes (*S. epidermidis* e *P. Aeruginosa*) grew slowly and, finally, other species survived only in lipid alone (*E. cloacae*). The authors concluded that lipid inclusion in PN represented a lower variability (3.3%) than glucose concentration, microbial species, and the interaction between microbe and infusion (respectively 5.8% vs. 35.3% vs. 4.4%). They found also that the growth of microbes, such as *C. albicans*, *E. coli*, or *S. epidermidis*, was not significantly related to the presence of lipid in PN at fixed glucose concentrations. Concerning the metanalysis that examined growth ratio (GR) variations during inclusion or addition of LE to PN, in one of them, the isocaloric exchange of glucose for lipid was not associated with an overall significant reduction in GR. Furthermore, another meta-analysis demonstrated that the lipid enrichment (1 Mcal) to lipid-free PN led to an overall non-significant rise in GR. Finally, in the last metanalysis, increasing the rate of non-protein energy as lipid in PN was associated with an overall non-significant decrease in GR. The authors concluded that the presence of lipids in PN is only one of several factors that may influence microbial growth in PN. Furthermore, regarding advice that limit the time of infusion of LEs from a single container to 24 h, or 48 h for lipid-free PN, the authors suggested that the duration of PN infusion from a single container should take into account several aspects, such as the glucose concentration, microbial species, interaction between microbe and lipid composition of the infusate, and it should be weighted based on microbial species which might contaminate PN [24]. From this meta-analysis, it is evident that in no case it is suggested to exclude lipids from the AIO bag to avoid infectious complications.

## 7. Conclusions

Regarding infectious complications related to CVCs (especially CRBSI) for patients in PN, there is no evidence that eliminating the lipidic component would prevent this kind of complication. This statement is not fully recognized by all clinicians, and nutritional training could highlight that the most important preventive measures remain full barrier precautions during insertion, aseptic handling of all connections, and dressing changes according to protocols developed and supervised by the nutritional team.

Nutritional education among clinicians is needed to clarify that LEs have evolved from being a primary source of energy for PN to becoming therapeutic substrates capable of targeted outcomes in critically ill patients.

## Data Availability

Not applicable.
